# The Use of Deep Learning-Based Gesture Interactive Robot in the Treatment of Autistic Children Under Music Perception Education

**DOI:** 10.3389/fpsyg.2022.762701

**Published:** 2022-02-10

**Authors:** Yiyao Zhang, Chao Zhang, Lei Cheng, Mingwei Qi

**Affiliations:** ^1^College of Art and Communication, Beijing Normal University, Beijing, China; ^2^School of Theater, Film and Television, Communication University of China, Beijing, China; ^3^School of Art, Ludong University, Yantai, China; ^4^Department of Music, Dalian Arts College, Dalian, China

**Keywords:** educational psychology, deep learning, artificial intelligence technology, gesture interactive robot, music perception education

## Abstract

The purpose of this study was to apply deep learning to music perception education. Music perception therapy for autistic children using gesture interactive robots based on the concept of educational psychology and deep learning technology is proposed. First, the experimental problems are defined and explained based on the relevant theories of pedagogy. Next, gesture interactive robots and music perception education classrooms are studied based on recurrent neural networks (RNNs). Then, autistic children are treated by music perception, and an electroencephalogram (EEG) is used to collect the music perception effect and disease diagnosis results of children. Due to significant advantages of signal feature extraction and classification, RNN is used to analyze the EEG of autistic children receiving different music perception treatments to improve classification accuracy. The experimental results are as follows. The analysis of EEG signals proves that different people have different perceptions of music, but this difference fluctuates in a certain range. The classification accuracy of the designed model is about 72–94%, and the average classification accuracy is about 85%. The average accuracy of the model for EEG classification of autistic children is 85%, and that of healthy children is 84%. The test results with similar models also prove the excellent performance of the design model. This exploration provides a reference for applying the artificial intelligence (AI) technology in music perception education to diagnose and treat autistic children.

## Introduction

The improvement of living standards of people has raised the requirements of people for spiritual life. The pursuit of material and spiritual civilization is also the internal driving force of social progress (Lim, 2019; Vaisberg et al., 2019). The mode of information dissemination is moving from the multimedia era to the all-media age. People are no longer satisfied with the text, sound, and image information based on auditory and visual perception, but continue to experience the complete media information of multiple perception channels based on sound, vision, and touch (Wu and Wu, 2017; Zheng et al., 2018). As a combination of art and entertainment, music can become a means and medium for emotional expression of people and then show the thoughts, feelings, and real-life experience of the creator (Qian et al., 2018; Chen, 2019). People perceive music mainly through hearing, but the emotional information contained in music is not limited to the auditory environment. The ancients demonstrated their feelings for music through sword dance and showed shocking auditory and visual experience through the coordination of hands, eyes, footsteps, and body (Rogoza et al., 2018; Wu and Song, 2019). People use advanced instruments and technologies to obtain wonderful visual, auditory, and emotional effects in modern life, such as music night and music fountain (Wu et al., 2019; Wu W. et al., 2020). Hence, since ancient times, people have interacted their desires with various perceptual methods in multiple ways to obtain the comprehensive perception of hearing, vision, and touch, and finally realize the emotion of synchronous interaction between people and music (Wu Y. J. et al., 2020; Yuan and Wu, 2020).

People with normal hearing hope to experience music synchronously and interactively through multiple perceptual channels of hearing, vision, and touch to further increase their immersion in music perception. Hearing-impaired people are also eager to experience music emotion, receive music education, and play music even when they cannot “listen” to music (Steel et al., 2020; Hooft et al., 2021). The research results of modern psychology show that music perception is a cooperative cognitive activity of multiperception system, which is not limited to hearing. Visual and tactile perception can perceive music through synesthesia under appropriate stimulation (Smit and Milne, 2021). The brain part of hearing-impaired people dealing with tactile information is the same as that of ordinary people dealing with auditory information (Baker et al., 2020). It means that hearing-impaired people can experience music like ordinary people with the touch of their body skin. Therefore, assisting or replacing touch can bring the experience of music perception to special groups and make them share the emotions psychologically and physiologically (Sorati and Behne, 2020; D’Onofrio and Gifford, 2021). Childhood autism is a developmental disorder in early childhood, involving sensory perception, emotion, language, thinking, action, and behavior. It is also the most common and typical developmental disorder. Some studies have proposed to treat children with autism through music; hence, this exploration is considered as the research goal to study the treatment and diagnosis of children with autism.

Music therapy is a comprehensive interdisciplinary subject integrating musicology, psychology, and medicine. Music therapy intervention on individuals depends on the four basic music functions, namely, physiological resonance, psychological empathy, social integration, and aesthetic sharing. The existing traditional treatment methods based on the four functions mainly include receptive music therapy, recreative music therapy, and impromptu music therapy. The effect of music therapy is widely recognized and accepted. However, there are still some shortcomings in its existing technical means, such as inaccurate targeting (e.g., lack of pathological pertinence and ignoring individual differences), time-consuming and laborious (e.g., high labor cost and limited site), low professionalism (i.e., unsystematic and objective efficacy evaluation indicators), and privacy disclosure. With artificial intelligence (AI) empowerment, the music therapy technology has made innovative development during diagnosis, treatment, and evaluation. The recurrent neural networks (RNNs) are used to optimize music therapy and improve the problem-solving ability of music therapy.

This exploration aims to improve the effectiveness of diagnosing and treating children with autism. According to the relevant theories of educational psychology, a music-teaching classroom with gesture interaction is constructed based on RNN, and different music perception treatment projects are used to treat autistic children. In the course, gesture interactive robots are used to teach music to autistic children, making students feel the charm of music through vision and realizing the treatment of autism through music. During the experiment, electroencephalogram (EEG) signals are used to detect the physiological state of children, and the RNN algorithm is used to identify and analyze the EEG signals of autistic children who are receiving different music perception treatment projects. The classification accuracy of the model and the course effect on the diagnosis and treatment of autistic children are verified. The research contribution is to provide a reference for the research on AI technology in music perception education and the diagnosis and treatment of autistic children. Besides, this exploration can provide a thinking path for the path and form of psychological change of children in music therapy.

The innovation is to apply RNN technology to the music perception treatment project for autistic children and try to treat autistic children through human-computer interaction (HCI). The research process is as follows. First, the status of this research is analyzed, and relevant problems and solutions are put forward. Then, the theories and methods involved in the research are introduced. Next, the research model and research framework are designed based on relevant research theories. Finally, the design method is tested to verify its effectiveness in dealing with the related problems.

## The Application of Artificial Intelligence Technology in the Education of Children

### Recent Related Work

Worldwide scholars have conducted extensive research on the application of AI technology and music perception education. Rodgers et al. (2021) studied the digital transformation effect of AI-based facial and music bioidentification technology on cognitive and emotional state of customers, and how these effects affect their behavioral responses in value creation. The participants experimented with different music types (enhanced by music recognition and bioidentification technology). The results show that the emotion caused by music recognition and bioidentification technology plays an intermediary role in cognitive and behavioral intention. This exploration can help understand the relationship between cognition and emotion induced by AI-based facial and music bioidentification systems in shaping the behavior of customers. Rahman et al. (2021) studied the relationship between music and emotion and introduced the emotional calculation into the physiological signal analysis. The data of skin electrical activity, blood volume pulse, skin temperature, and pupil dilation were collected. The neural network was used to identify and evaluate the subjective emotions of participants to verify the application effect of music therapy in mental health nursing. Koempel (2020) studied the application of AI in the music industry, which defines music copyrights to judge whether there is a copyright problem by intelligently identifying music content. Sharda et al. (2019) studied the application of music therapy for autistic children. Music therapy is used to experience music by trained therapists to promote health. It can improve the communication skills of autistic children and other abilities of children. Matokhniuk et al. (2021) studied the therapeutic effect of music therapy in dealing with the psychological characteristics of anxiety in adolescents. Under increasing anxiety, music therapy was adopted to explore the changes in anxiety level of teenagers in the psychological situation. The results show that the psychological correction of music therapy is effective in the work of psychologists.

Given the above analysis, AI technology can be used to build a more sound music curriculum system. Music therapy in the treatment of autistic children is a crucial exploration. However, there are less studies on the combination of the two. Hence, an AI-based human-computer interactive music classroom is established to treat autistic children through gesture interactive robots combined with music perception therapy. The mental state and treatment effects of children are analyzed by monitoring their EEG signals to provide a reference for research in related fields.

### Educational Psychology

Educational psychology is an applied discipline, and its theoretical and practical research has been proved to be essential. It marks the changes of educational model of China from tradition to science. The research on educational psychology took shape in ancient China, but the research on learning psychology of students starts late in China (Sahli et al., 2019; Vaisberg et al., 2019). Psychologists and educators of music education have been studying the development process of learning motivation of students, psychology and cognitive ability, and doing scientific research in other aspects, showing a systematic trend. Scientific research aims to seek diversity in students, teachers, social and family environments, and other aspects (Besedová et al., 2019; Zhang et al., 2021). Some achievements have been made in the research on educational psychology and its branch of learning psychology. However, some deficiencies in the research methods of music psychology in China still exist. Music education is a crucial part of aesthetic education in the overall development of mankind and is effective in cultivating cognitive, emotional, and behavioral abilities of students (Choi et al., 2018; Parkinson et al., 2019). Music is studied through learning psychology. Teaching methods suitable for learning psychology and culture of students are selected to improve the interest of learners in learning music, enrich teaching methods, respect individual differences, and achieve music training of people. It is crucial for improving the quality of music teaching in primary schools and cultivating high-quality talents.

### The Basic Theory of Deep Learning

**FIGURE 1:**
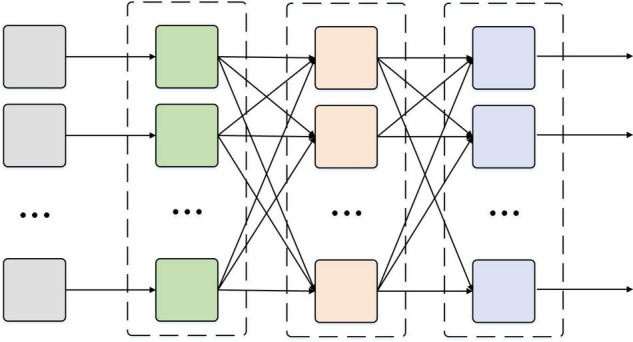
RNN structure diagram.

**FIGURE 2:**
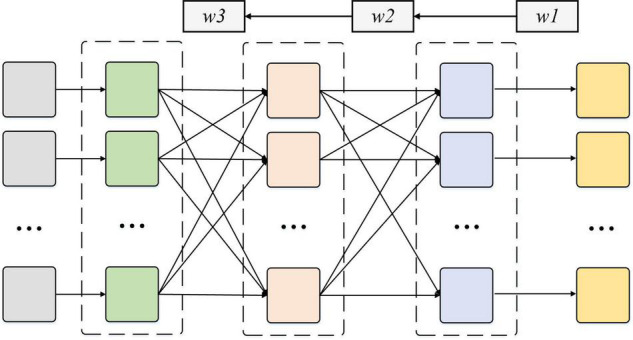
Structure diagram of multilayer reverse neural network.

The RNN is quite effective for data with sequence characteristics. It can mine temporal information and semantic information in data. Therefore, RNN is widely used in time-series-related data processing. According to the characteristics of EEG signals, the RNN algorithm is used as the main research algorithm. The basic structure of the RNN model is the input layer, hidden layer, and output layer from left to right. The data are input from the leftmost input layer and output from the rightmost output layer. Layers are fully connected. RNN is actually a non-linear input-output mapping (Andréanne et al., 2018; Kondo et al., 2018). The input value of network is taken as the independent variable of the non-linear function. The output prediction value of network is considered the dependent variable of non-linear function. When the input and output nodes are considered *a* and *b*, RNN represents the functional mapping relationship of *a* and *b*. Figure 1 shows the decoupling strand of RNN, and Figure 2 shows the structure of a multilayer reverse neural network.

In the RNN learning algorithm, the input layer is set as *a*_*i*_, the middle hidden layer is set as *b*_*j*_, and the output layer is set as *c*_*k*_. The connection weight between the input layer and the hidden layer is *f*_*ij*_, and the connection weight between the hidden layer and the output layer is *g*_*jk*_. The thresholds of the output and hidden layers are recorded as *d*_*l*_ and *e*_*m*_, respectively, and the expected output value of the output layer is *h*_*n*_.

In the RNN model, the neuron is net, and its function form reads:


(1) $$n\,e\,t=\sum_{i=1}^{n}g_{i}\,a_{i}-e$$

The activation function form of RNN reads:


(2) $$g\,\left(a\right)={\left(1+e^{-k\,a}\right)}^{-1}$$

The derivative form of the RNN activation function reads:


(3) $$g^{\prime }\,\left(a\right)=g\,\left(a\right)\,\left(1-g\,\left(a\right)\right)$$

The principle of RNN is to divide the learning process into the forward propagation of signal and backward propagation (BP) of error signal.

The forward propagation process of BP neural network signal:

The output from the input layer to the hidden layer is:


(4) $$b_{j}=g\,\left(n\,e\,t_{j}\right)=g\,\left(\sum_{i=1}^{n}p_{i\,j}\,a_{i}-e_{j}\right)$$

The calculation of neural unit *j* reads:


(5) $$n\,e\,t_{j}=\sum_{i=0}^{n}p_{i\,j}\,a_{i}$$

The output from hidden layer to output layer is:


(6) $$c_{k}=f\,\left(n\,e\,t_{k}\right)=f\,\left(\sum_{i=1}^{n}g_{i\,k}\,b_{j}-d_{l}\right)$$

The calculation of neural unit *k* reads:


(7) $$n\,e\,t_{k}=\sum_{j=0}^{n}g_{i\,j}\,b_{j}$$

Signal error BP process of BP neural network:

The error function reads:


(8) $$E=\frac{1}{2}\,\sum_{k=1}^{n}e_{k}^{2}$$

Error function of hidden layer reads:


(9) $$E=\frac{1}{2}\,\sum_{k=1}^{n}{\left[h_{n}-f\,\left(\sum_{j=0}^{n}g_{i\,j}\,b_{j}\right)\right]}^{2}$$

Error function of the input layer reads:


(10) $$E=\frac{1}{2}\sum_{k=1}^{n}[h_{n}-f{\left(\sum_{j=0}^{n}g_{i\,j}p\left(\sum_{i=0}^{n}p_{i\,j}a_{i}\right)\right]}^{2}$$

Equation 7 shows that the error function is a function of the connection weight *f*_*ij*_ between the input layer and the hidden layer, and the connection weight *g*_*jk*_ between the hidden layer and the output layer. Then, changing the connection weight can reduce the error function value *e*. The ultimate goal is to reduce the weight continuously. Then, the weight should be reduced accordingly, i.e.,


(11) $$\Delta \,p_{i\,j}=-\eta \,\frac{\partial E}{\partial p_{i\,j}}$$

(12) $$\Delta \,g_{j\,k}=-\eta \,\frac{\partial E}{\partial g_{j\,k}}$$

The above Equations 11, 12 reflect the learning rate of BP neural network.

Next, it is essential to define the error signal, and then adjust the corresponding weights and thresholds, so that they can show the same gradient changes.


(13) $$\delta_{j\,k}=\sum_{k=1}^{k}\left(h_{k}-c_{k}\right)\,c_{k}\,\left(1-c_{k}\right)$$

(14) $$\delta_{j\,k}=\delta_{j\,k}\,g_{j\,k}\,b_{j}\,\left(1-b_{j}\right)$$

The equation for adjusting the weight of the hidden layer reads:


(15) $$\Delta \,p_{i\,j}=\eta \,\delta_{i\,j}\,a_{i}$$

The equation for adjusting the weight of the output layer reads:


(16) $$\Delta \,g_{j\,k}=\eta \,\delta_{j\,k}\,b_{i}$$

The equation for adjusting the threshold reads:


(17) $$\Delta \,d_{l}=-\eta \,\delta_{i\,j}$$

(18) $$\Delta \,e_{m}=-\eta \,\delta_{j\,k}$$

### Gesture Interactive Robot

The HCI refers to the communication and interaction between humans and robots. Traditional HCI usually includes reading the state of robots through the display interface. Humans control robots through clocks, joysticks, or keyboard buttons. Such traditional operation method is often incompatible with human natural knowledge, and robot operation usually requires professional training. The traditional HCI methods have become increasingly difficult with the complexity of robot functions. Operators often cannot complete robot tasks quickly and skillfully, so HCI methods in line with the intuition of ordinary people are becoming increasingly popular. Natural HCI does not require other complex equipment, and operators do not need to receive complex training.

Human-computer interaction can be achieved through existing intuition. The interaction between humans and natural computers significantly improves the control ability and work efficiency of humans. As the main form of human natural language, gesture has become the first choice of HCI. The commonly used gesture recognition methods can be divided into data glove-based methods and vision-based methods according to different sensor types. The former has the advantages of the small data amount and good real-time performance. However, its disadvantages are also obvious. The operator must carry the corresponding data acquisition equipment, which will bring inconvenience and high cost. The advantages of the latter are no need to be implemented, such as low cost and easy to use. The disadvantages are the low recognition rate and complex algorithm.

### Music Perception Education

Music is closely related to various human activities. It is very crucial for human beings because of its emotional feedback effect. Music can trigger the electrophysiological activities of the brain and express music emotion in the form of brain waves (Gage and Baars, 2018; Nazarov, 2021). The cognition of music of the brain is a complex process, and the cognitive effect is mainly manifested in the change of EEG waveform. The surface characteristics of EEG cannot well explain music emotion (Han et al., 2019; Solberg and Dibben, 2019). Thereby, massive researchers are gradually exploring the deep characteristics of EEG. The development of brain science and cognitive science has greatly promoted the research on the relationship between music and brain function.

Music can stimulate the release of some transmitters affecting brain function, thus promoting functional coordination among regions, because the central control of emotion is the hypothalamus of the human brain and the limbic system of the human brain and music. These areas directly act on the human body and can regulate emotion in both directions to help the treatment of physical and mental diseases. The temporal lobe region of the brain contains both the auditory central system of the brain and the pain central system. Music can alleviate pain to a certain extent, because music stimulation can make the auditory center more active to inhibit pain. The right brain mainly dominates musical activities. Intervention and perceptual music therapy can improve brain function, coordinate the balance between hemispheres and rights in the left brain, accelerate intelligence development, improve cognitive ability, and provide special education for children with mental diseases and autism.

## Research Model and Research Framework

### Signal Acquisition

The non-implantable method is adopted to collect brain waves, which has less harm to the human body with a simple operation.

(1) Selection of subjects:

Selection of experimental subjects: experimental subjects are children aged 3–12 years. They have no serious hyperactive behavior, no other serious diseases, no vigorous exercise, and no cold and fever before the experiment. Children aged below 12 are in a critical period of physical, mental, and cognitive development. Autistic children have the best physiological advantages of mental energy and physical recovery during this period. Music perception education and therapy can use this advantage. A total of 20 autistic children are investigated. They are recently interviewed by the department of psychology of the cooperative hospital. The data obtained are only used in this experiment, and the subjects have authorized the data collection and use.

Autistic children may have aggressive behavior. Intelligence tests are conducted to screen several children who meet the requirements. It is essential to make children feel the warmth of psychologists mainly through dialogs. After the standard screening, 10 autistic children are selected as the main subjects, and healthy children are taken as the control group. Then, music experience and needs of each experimental subject are investigated and counted, mainly using the questionnaire, interview, and literature analysis methods. The most popular songs, the least popular songs, and unknown song styles in each theme are counted and recorded. Referring to the selection methods of these records and music works for the same, four types of music are finally determined, namely, sad music, happy music, soft music, and no music environment.

(2) Selection of music works: appropriate music must be selected to make music perception therapy have a palliative effect on autistic children. Music is a kind of sound art. For autistic people, not all music is suitable for curing the disease. First, the scope of music should not be too large, and the music should be soft, without extreme tonal changes and no sound disharmony. Next, everyone is independent, and the sensitivity to music style is quite different. Hence, while meeting the universal standards of autism treatment, music must be selected for each theme. Thereby, the team members design an online questionnaire to collect and classify massive information about the interest of children in music and music types. The following description of music selection and experiment is found. Music is selected according to the interest of each child. Experiments show that children can more effectively follow the music to respond to body and facial expressions when hearing their favorite music, making the treatment more effective.

**FIGURE 3:**
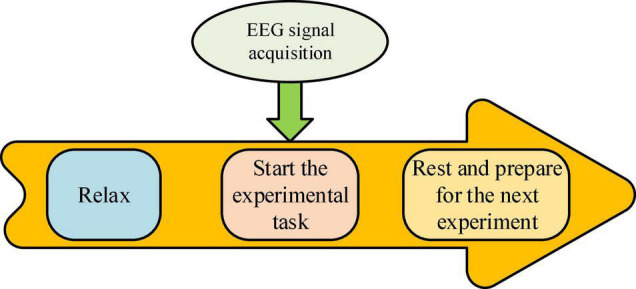
Experimental process.

The music work selection emphasizes rhythm. Autistic children often lag behind in some aspects, so choosing a musical instrument as an experimental environment to produce a strong and simple rhythmic sound will usually produce good results. Children aged below 12 are also more likely to accept unique music styles. The choice of works must meet the own needs of children. The music should not be too long. Otherwise, auditory fatigue will affect the traditional EEG response during the experiment. Appropriate rest during the experiment is necessary to reduce the fluctuations caused by negative emotions. Figure 3 shows the specific process of EEG acquisition. It reveals that the acquisition of EEG requires the tester to go through the three stages, and then the corresponding data results can be collected.

### An Improved Algorithm for Electroencephalogram Denoising

The EEG signal is a kind of bioelectric signal from the surface of the human scalp. It has the similarity of bioelectric signals, namely, small amplitude, low frequency, and strong randomness, so it is easy to be affected by all kinds of noise during acquisition. There are three types of noise, such as basic drift, power frequency interference, and eye interference. In basic drift, the displacement, breathing, movement, and human spirit of the electrode patch are not specific, which may lead to deviation from the basic position of the waveform of the normal brain. The data in different degrees become larger or smaller and move downward and upward as a whole. It can be found that over time, this phenomenon is called basic drift in the acquisition process of real-time display of brain waveforms. Basic drift is a low-frequency interference that can be eliminated in the frequency range of 0.15–0.3 Hz by the normal decoupling method, occasionally reaching 1 Hz. In power frequency interference, during data acquisition, energy-consuming equipment, such as computers, power adapters, and electric lamps, will produce power frequency interference and reduce the signal-to-noise ratio of EEG signals. Power frequency interference is usually manifested as small defects in EEG. Sometimes, the interference is too large and may even drown a useful weak signal. The interference frequency range of power frequency is about 50 Hz. Hence, the experimental environment should be open, such as the school playground. In electric eye interference, although subjects can collect EEG signals with their eyes closed, they cannot avoid turning their eyes. It is essential to avoid artificial products with eye flicker caused by interference, contraction, and relaxation of skin and respiration caused by interference, which will affect the measurement results. The electrode is attached to the scalp. The movement of the eyes can change the electromagnetic field of the skin around the eyes. The skin can pull the change of the skin around the skin and then affect the electromagnetic field around the electrode. Eye movement brings multiple difficulties to EEG acquisition. The greatest difficulty in EEG acquisition is physiological interference, which is difficult to eliminate by traditional solutions.

Algorithm principle: the first step of EEG signal denoising is to decompose the mixed EEG into the original modal function, and the empirical modal decomposition principle is used. The second step is to transform each original modal function by the Hilbert transform, obtain the corresponding Hilbert spectrum, and extract the noise component using a computer. The third step is to remove the spectrum with noise component, and the remaining original modal function components are summed to obtain the denoised EEG signal.

The principle of empirical mode decomposition is to decompose the unstable EEG signals into modal functions with certain modes by using computers adaptively. First, the maximum and minimum values of the input EEG signal *a*(*x*) are solved. The maximum value is recorded as *a*(*x*_*i*_), and the minimum value is recorded as *a*(*x*_*j*_). Next, the average of the maximum and minimum values is calculated and recorded as *b*(*x*):


(19) $$b\,\left(x\right)=\frac{a\,\left(x_{i}\right)+a\,\left(x_{j}\right)}{2}$$

Next, a new data sequence *c*(*x*) is obtained by subtracting the average EEG signal from the initial input EEG signal:


(20) c⁢(x)=a⁢(x)-b⁢(x)

The first two steps are carried out until the conditions of the natural modal function are met to obtain *c*_1_(*x*):


(21) $$d\,\left(x\right)=a\,\left(x\right)-c_{1}\,\left(x\right)$$

Then, the noiseless signal is obtained by linear addition.


(22) $$a\,\left(x\right)=\sum_{i=1}^{n}c\,\left(x\right)+d\,\left(x\right)$$

*c*(*x*) represents the *i*-th inherent mode component, and *d*(*x*) can be a constant or a monotone function.

After the Hilbert transform for each inherent mode component, the equation of the initial EEG signal reads:


(23) $$a\,\left(x\right)=\sum_{i=1}^{n}e_{i}\,\left(x\right)\,\exp\left(j\,\int f_{i}\,\left(x\right)\,dt\right)$$

_*e_i_ (x)*_ represents the analytical function, and the equation of the marginal spectrum reads:


(24) $$g\,\left(f\right)=\int_{-\infty }^{+\infty }H\,\left(f,x\right)\,dx$$

**FIGURE 4:**
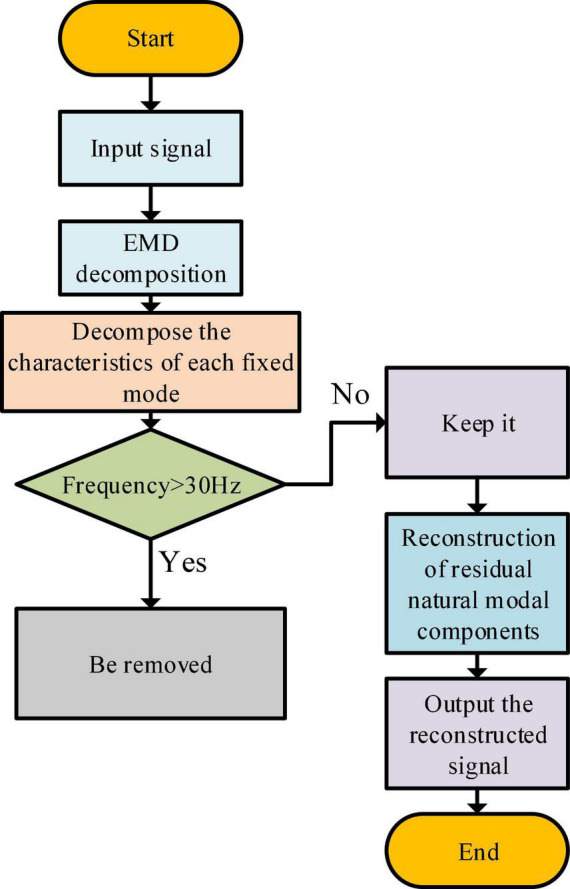
Denoising flowchart.

The above functional changes have high reliability and can obtain denoised EEG signals. Figure 4 shows the specific denoising process.

### Feature Extraction and Classification Based on Deep Belief Network

The energy of the proportional frequency band is selected based on the features. Each rhythm is abnormal EEG due to large distribution. Autistic patients are visually diagnosed to analyze the collected EEG data for scientific research and clinical application. The energy ratio of the selected frequency band is one of the same speed characteristics. First, an intercepted EEG data sample is selected. The range of values corresponding to the frequency band of each EEG frequency is determined. The energy of any frequency can be calculated from the wave coefficient, so the harmonic transformation of the wave packet must be conducted first. The band energy of each frequency is calculated through the converted wave coefficient.

## Results Analysis

### Experimental Environment

Matlab R2018a is used to build the software environment of the experiment, and the hardware environment is designed to collect the EEG data of subjects. The EEG signal collection equipment used is the neuroscan EEG acquisition and analysis system, a new generation of authoritative amplifier in the research on electrophysiological amplifiers. Table 1 shows the software and hardware settings of the experiment.

**TABLE 1:** Software and hardware settings.

Items	Equipment status
Software	Matlab R2018a Neuroscan EEG acquisition and analysis system
System	Win10
CPU	i7-11800H
GPU	GeForce GTX 3050 Ti
Program	Python 3.9
Hard disk capacity	16 GB DDR4+512 GB SSD

### Experimental Dataset and Preprocessing

Relevant data need to be collected as a dataset to test the effectiveness of the design algorithm. Therefore, the EEG signals of 10 autistic children and 10 normal children under music perception treatment are collected. The data are collected in different stages, and the EEG signals of children with different courses of treatment are collected. In the neural network test, the collected data are divided into training set and test set according to the ratio of 5:1 to realize the training and testing of the model. The experimental results show the effectiveness of the design method.

### Hyperparameter Setting

According to the selection of entropy characteristics of sliding samples, entropy is a parameter that can quantitatively describe unstable physical systems or signals. The entropy of samples has good coherence and anti-noise performance and is suitable for short data analysis. For example, in the multiscale entropy analysis of EEG data of autistic children, the sample entropy algorithm is adopted to calculate the entropy, but it is limited to the complete analysis of short-term EEG information. At the theoretical level, the entropy of samples represents the entropy estimation of fixed data in time series. However, the EEG waveforms of subjects listening to music or resting are different over time and are non-fixed signals. Therefore, it is suggested to analyze the long-term EEG data in a time range. In each analysis window, it can be assumed that the processed data are relatively stable to study the average entropy characteristics of sliding samples to reflect the whole brain activity under continuous complex background conditions.

The hyperparameter setting of the RNN algorithm needs to be adjusted to better analyze the characteristics of EEG signals. The parameters are constantly adjusted to compare the output research results to obtain the optimal super parameter setting. Table 2 shows the settings of some hyperparameters.

**TABLE 2:** Hyperparameter setting of RNN.

Parameters	Value	Parameters	Value
Learning rate	0.2	Activation function	Sigmoid function
Maximum training times	1000	Weight change increment	1.5
Training required accuracy	0.00001	Weight change reduction	0.7
Minimum gradient requirements	1.00E-10	Initial weight change	0.07
Show training iteration process	500	Maximum value of weight change	100
Loss function	Exponential Loss	Gradient descent algorithm	Nadam algorithm

### Denoising Simulation Results

**FIGURE 5:**
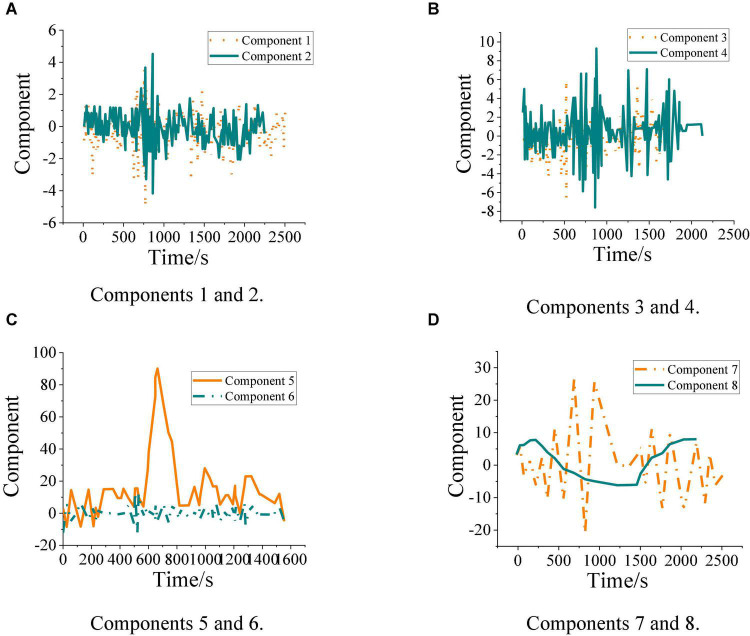
Time-domain diagram of each natural mode component. **(A)** Components 1 and 2. **(B)** Components 3 and 4. **(C)** Components 5 and 6. **(D)** Components 7 and 8.

The performance of the model decomposition target signal is tested to verify its ability to process the related data and study the effectiveness of the design model. Figure 5 shows the result of decomposing the target signal frequency.

Figure 5 shows that the signals of natural modes a–d belong to the signals of high-frequency components, so these original signals belong to noise signals. Four groups of signals with lower frequency components than the original signal are obtained by decomposing the high-frequency signal through the model. This proves that the designed model can effectively reduce the noise signal. In practical application, it can decompose and process the actual EEG signal, eliminate the noise in the EEG signal, and improve the accuracy of model recognition. Therefore, it can effectively analyze the information contained in brain signals and help researchers understand the psychological status of testers.

**FIGURE 6:**
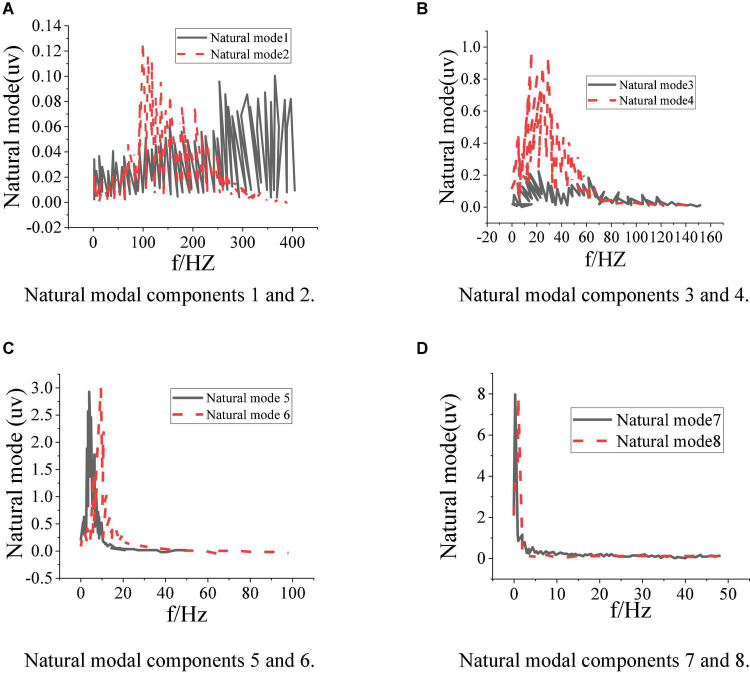
Modal spectrum of each inherent brain signal. **(A)** Natural modal components 1 and 2. **(B)** Natural modal components 3 and 4. **(C)** Natural modal components 5 and 6. **(D)** Natural modal components 7 and 8.

Figure 6 shows the identification results of the inherent brain signal modal spectrum of design model.

Figure 6 presents that the spectrum diagram contains many spectra higher than 30 Hz, while the frequency of brain waves of the normal human brain is below 30 Hz. It shows that part of the signals identified by the model are noise signals, while the signals identified in (c) and (d) are normal EEG signals. Hence, the recognition results of the model of EEG signals need to be strengthened. This phenomenon may be due to the noise of corresponding frequency in the environment, making the recognition results of the model wrong. It suggests that the designed model still needs to be further optimized to improve the ability to deal with problems and cope with the more complex experimental environment.

### Music Perception Results of Electroencephalogram in Autistic Children

**FIGURE 7:**
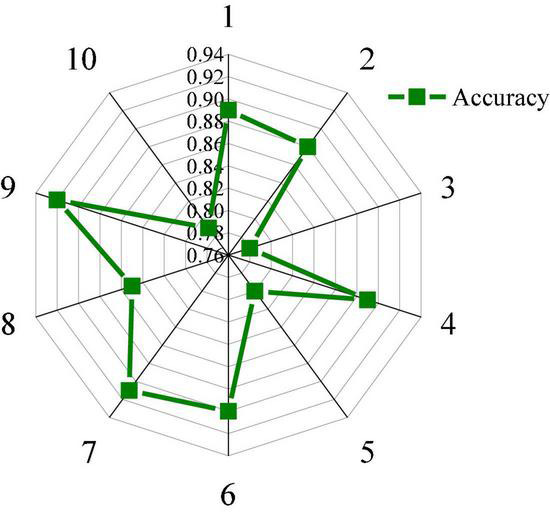
Classification accuracy of 10 autistic children.

The accuracy of music perception of autistic children under music perception treatment is tested. Figure 7 shows the classification and statistics results of music perception accuracy of 10 autistic children.

Figure 7 shows that the classification accuracy of the model for music perception of autistic children under music perception therapy fluctuates in a large range, indicating that different autistic children have different sensitivity to music. The classification accuracy of music perception of most autistic children is between 75 and 94%, the highest classification accuracy is 92%, the lowest classification accuracy is 78%, and the average classification accuracy is 85%. Overall, the designed model is accurate in classifying music perception of autistic children, but the classification effect is poor for some special children. Therefore, the model performance needs further optimization.

### Music Perception Results of Electroencephalogram in Healthy Children

**FIGURE 8:**
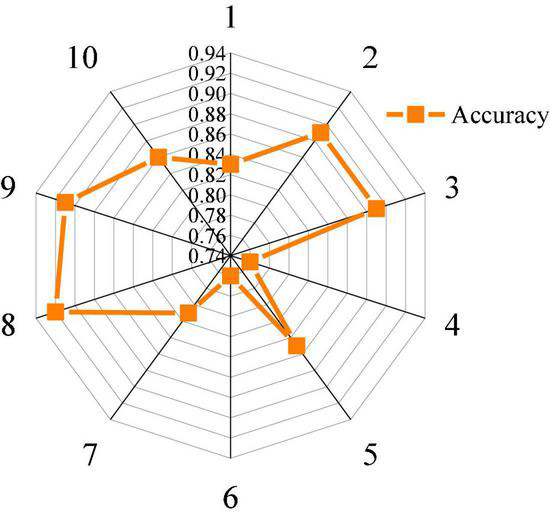
Classification accuracy of 10 healthy children.

The accuracy of music perception of healthy children under music perception treatment is tested. Figure 8 shows the results of classification and statistics of music perception accuracy of 10 healthy children.

Figure 8 suggests that the accuracy of EEG classification in healthy children is also different. In this experiment, the highest accuracy of model classification is 92%, the lowest accuracy is 76%, and the average accuracy is 84%. It also proves the stability and feasibility of the classification accuracy of deep belief network (DBN). Overall, the classification results of the designed model for music perception of healthy children are accurate, and there may be classification errors for some special objects. Compared with the test results in Figure 7, it can be proved that the designed model has a better analysis effect on dealing with EEG problems of autistic children. Therefore, it can be used to analyze the changes in the psychological status of autistic children in music therapy.

### Performance Comparison

The performance of the designed algorithm is compared with similar models and different test sets to verify its effectiveness. The algorithms used include long short-term memory (LSTM) network, convolutional neural network (CNN), and support vector machine (SVM) algorithms. Table 3 shows the results.

**TABLE 3:** Performance comparison.

Algorithm	Autistic children	Healthy children
CNN	57.01%	60.7%
LSTM	74.45%	64.87%
SVM	74.76%	52.87%
The designed algorithm	85.45%	84.01%

Table 3 shows that the experimental results of the designed algorithms are superior to similar algorithms, and the recognition accuracy of different experimental datasets is about 85%. The experimental results of similar algorithms are obviously different from the test results of different datasets, which shows that the designed algorithm can better adapt to the changing experimental conditions to get better experimental results.

### Discussion

In conclusion, different types of experiments verify the effectiveness of the design model in dealing with related problems and provide a certain reference for teaching autistic children and promoting music therapy. In the test experiment, the highest model classification accuracy is 92%, the lowest accuracy is 76%, and the average accuracy is 84%. Comparative experiments show that the designed method has a good effect in dealing with similar problems. Therefore, the designed model can well detect the psychological state of autistic children, and further diagnose and treat autistic children in the curriculum. The research provides a reference for the treatment and diagnosis of autistic children and points out a different development path for the future education of children. The designed method can provide an effective reference for the teaching and disease treatment of autistic children and help readers understand the form and path of the impact of music therapy on psychology of children.

## Conclusion

This exploration aims to apply music perception to the diagnosis and treatment of autism and effectively help autistic children communicate with others and express their emotional interaction ability in social activities. The gesture interactive robot is designed based on the related concepts of educational psychology and deep learning technology. Moreover, EEG is used to perceive the condition and treatment effect of autistic children. In the research process, RNN is used to realize the interactive communication of gesture interactive robots. Then, the brain signals of autistic children before and after the experiment are collected and analyzed based on the brain signal denoising algorithm. Data features are extracted through DBN. The experimental results show that different people have different perceptions of music, but these differences fluctuate in a certain range. The classification accuracy of the designed model is about 74–94%, and the average classification accuracy is about 85%.

However, there are still some research deficiencies. The application mode and course mode of HCI robots in the actual classroom need to be further studied to improve its applicability. At present, the recognition accuracy of the research model fluctuates greatly, and the model needs to be further optimized. Moreover, the research is still in the preliminary stage, so the number of samples that can be studied is small, which cannot accurately reflect the practical application results of the model. In the follow-up research, the number of samples will be expanded, and the designed algorithm and HCI course mode will be further optimized to further improve the applicability and effectiveness of the research results.

## Data Availability Statement

The raw data supporting the conclusions of this article will be made available by the authors, without undue reservation.

## Ethics Statement

The studies involving human participants were reviewed and approved by the Ethics Committee of Beijing Normal University and Dalian Arts College. The patients/participants provided their written informed consent to participate in this study. Written informed consent was obtained from the individual(s) for the publication of any potentially identifiable images or data included in this article.

## Author Contributions

All authors listed have made a substantial, direct, and intellectual contribution to the work, and approved it for publication.

## Conflict of Interest

The authors declare that the research was conducted in the absence of any commercial or financial relationships that could be construed as a potential conflict of interest.

## Publisher’s Note

All claims expressed in this article are solely those of the authors and do not necessarily represent those of their affiliated organizations, or those of the publisher, the editors and the reviewers. Any product that may be evaluated in this article, or claim that may be made by its manufacturer, is not guaranteed or endorsed by the publisher.
